# Gonadotropin-Releasing Hormone Analog Cotreatment for the Preservation of Ovarian Function during Gonadotoxic Chemotherapy for Breast Cancer: A Meta-Analysis

**DOI:** 10.1371/journal.pone.0066360

**Published:** 2013-06-21

**Authors:** Chuan Wang, Minyan Chen, Fangmeng Fu, Meng Huang

**Affiliations:** 1 Department of General Surgery, Fujian Medical University Union Hospital, Fuzhou, China; 2 Union Clinical School, Fujian Medical University, Fuzhou, China; 3 Fujian Center for Disease Control and Prevention, Fuzhou, China; Dartmouth, United States of America

## Abstract

**Objective:**

To determine by meta-analysis whether gonadotropin-releasing hormone analog (GnRHa) cotreatment accompanying chemotherapy for breast cancer protects ovarian function.

**Methods:**

Randomized controlled trials (RCTs) comparing GnRH cotreatment with chemotherapy alone in premenopausal women were collected by electronic and manual searches of Pubmed, MEDLINE (OVID), CENTRAL (The Coehrane Central Register of Controlled Trials), CBM, CNKI, VIP and Wanfang data bases. All the data was analyzed by Stata 11.2.

**Results:**

Seven studies with a total of 677 participants met the inclusion criteria. The outcome of meta-analysis implied that, compared with adjuvant chemotherapy alone, the number of patients with resumption of spontaneous menstruation was statistically greater in the GnRH cotreatment patients (OR 2.83; 95% CI, 1.52–5.25).

**Conclusions:**

Evidence from RCTs suggests a potential benefit of GnRH cotreatment with chemotherapy in premenopausal women, producing higher rates of spontaneous resumption of menses.

## Introduction

Breast cancer is the most common cancer among women, and is also common among reproductive age women. In 2011, the United States SEER data showed that approximately 6% of women with breast cancer are diagnosed before age 40 [1], and the probability of developing breast cancer before age 40 is nearly 1/200 [2]. Major advances in diagnosis and treatment of breast cancer have reduced mortality, and a majority of patients diagnosed with breast cancer will be long-term survivors. Because young women with breast cancer face challenges when considering future fertility, it is important to preserve ovarian function in cancer patients who must undergo chemotherapy.

Improved cancer survival rates are directly related to major advances in our understanding of cell division, multiplication, and growth, which has led to the development of more effective chemotherapeutic agents that selectively target rapidly dividing cells [3], [4]. However, few chemotherapeutic agents are cell type specific and they damage a wide range of rapidly dividing cell types, such as bone marrow, thymus, and gastrointestinal mucosa. Gonadal tissue is also highly sensitive to these agents, and pre-ovulatory follicles are particularly sensitive to alkylating agents [5], [6].

The majority of young patients with breast cancer receive systemic treatment with chemotherapy, hormone therapy, or both. These patients are at high risk of transient or permanent amenorrhea, and for those women who continue to menstruate or who recover their cycles, there is an additional long-term risk of premature ovarian failure (POF) [7]. Chemotherapeutic agents most likely to induce POF include cyclophosphamide, L-phenylalanine mustard, busulfan, and nitrogen mustard [8]. POF leads to symptoms of low estrogen, such as hot flashes, vaginal dryness, low libido, and osteoporosis that severely affect the quality of life. So a large number of experiments have been conducted to find ways to reduce ovarian damage or protect ovarian function. Embryo and oocyte preservation technologies are complex, expensive, and have a low success rate [9]. And before the collection both require stimulation of the ovaries to make a high levels of estrogen, which is a potential risk for hormone-dependent patients [10]. In addition, ovarian tissue cryopreservation and transplantation are still under investigation, and the potential risk of introducing cancer is not known. Compared to these methods, the GnRHa is a simple, viable, and low-cost alternative.

Theoretically, temporarily returning ovaries to an inactive state by inhibition of the pituitary-gonadal axis before chemotherapy might make the germ cells less susceptible to the effects of alkylating agents [11]. Originally, GnRHa were used for this purpose. In the 1980s, the effectiveness of a GnRHa during chemotherapy to preserve ovarian function was first demonstrated in rodents and monkeys [12]–[14]. Over the last 10 years, more than a dozen reports of small cohorts of women undergoing chemotherapy with concomitant GnRHa therapy have been published, but the results were inconsistent. Some prospective controlled studies of concomitant GnRHa and chemotherapy have demonstrated a decrease in the ovarian failure rate after chemotherapy compared with controls [15]. However, other studies have not shown a benefit [16]. The primary objective of our systematic review of the literature was to ascertain the effectiveness of adding GnRHa to chemotherapy for the preservation of ovarian function during chemotherapy for breast cancer.

## Materials and Methods

### Search Strategy

We searched for published trials in the electronic databases MEDLINE (OVID), PubMed, the Cochrane Central Register of Controlled Trials (CENTRAL) in the Cochrane Library, CBM, CNKI, VIP and WanFang data bases. We also did a manual retrieval of relevant academic conference papers, assemblies and dissertation. The search covers January 2000 to June 2012. At the same time, we screened the included articles and according to the referenced literature, further expanded our retrieval. The search syntax was tailored individually for each database but included the following main Medical Subject Headings (MeSH) and text words; gonadotropin-releasing hormone, chemotherapy, ovarian preservation, and breast cancer.

### Inclusion and Exclusion Criteria

Studies included in the meta-analysis met the following criteria: 1) Type of research had a randomized controlled design with the language limited to Chinese or English. 2) The research object was premenopausal women with a pathological diagnosis of breast cancer, with a detailed description of the patient′s basic characteristics. 3) Intervention included the GnRH group with GnRHa and chemotherapy treatment and the control group with chemotherapy alone, with no limit to the chemotherapy scheme and the GnRHa treatment. 4) Outcome measures were the incidence of not having premature ovarian failure. 5) Published research.

Studies excluded from the meta-analysis had the following criteria: 1) Controlled trials that were not randomized. 2) Nontherapeutic clinical research and animal experiments. 3) Publications in other than the Chinese and English language literature. 4) Studies with no related outcome measured. 5) Studies with a dropout rate of greater than 10%. 6) From repeated publication of studies, we took the latest, with the most complete data.

### Data Extraction and Quality Assessment

Two reviewers independently conducted data extraction and assessed the quality of each study for meeting the inclusion criteria. Data extraction included the descriptive characteristics of the study patients (gender, average age, chemotherapy scheme and estrogen receptors, etc.), the characteristics of the intervention (the dose, the time of administration, cycle and other treatment), outcome measures and the final results.

We assessed study quality using a four item method developed by Jadad and colleagues that evaluated the adequacy of randomization [17], concealment of randomization, blinding, and completeness of follow-up. When a study described the method of randomization correctly, it could get two points, but got one point if it only mentioned the randomization. We used the same principle for the blinding and completeness of follow-up. If the study mentioned the allocation concealment, it could get one point. There was a maximum score of seven points, and using the four evaluation criteria, the literature was divided into three levels. Class A - low bias with 5 to 6 points: met all the evaluation standards and the study was the least likely to produce all kinds of bias. Class B - a moderate bias with 3 to 4 points: partially met the quality standards and the possibility of producing a variety of bias was moderate. Class C - a high degree of bias with 1 to 2 points: not satisfying the quality standards, the study had a high possibility of a variety of bias.

### Statistical Analysis

Data management and statistical analysis were conducted using Stata 11.2 [18]. Where possible, data were extracted to allow for an intention-to-treat analysis, defined as including in the denominator the number of all originally included patients. If data from the trial reports were insufficient or missing, the investigators of the individual trials were contacted via E-mail for additional information.

For the meta-analysis, the number of participants in each group of the trial was recorded. This review used the odds ratio (OR) and 95% confidence interval (95% CI) as the effect size. First, we analyzed the clinical heterogeneity of the included trials. Second, for statistical heterogeneity we used the chi-square test with a 5% level of statistical significance. In addition, heterogeneity was quantified using the I^2^ test. If P>0.05, we considered that the included trials had homogeneity of data, and the pooled OR was estimated by the fixed-effects model (the Mantel-Haenszel method) [19]. Otherwise, if P<0.05 and I^2^ >50%, heterogeneity was found. We attempted to determine the possible source of heterogeneity and the random-effects model (the DerSimonian and Laird method) was employed [20].

Publication bias of literature was assessed using funnel plots and Egger’s test. An asymmetric plot suggests a possible publication bias and the P value of the Egger’s test less than 0.05 was considered representative of a statistically significant publication bias [21].

### Sensitivity Analysis

To determine whether any study of relatively poor quality was incurring undue weight in the analysis, we systematically removed the data and checked the pooled results for the remaining studies to see if they changed significantly.

## Results

### Search Results

**Figure 1:**
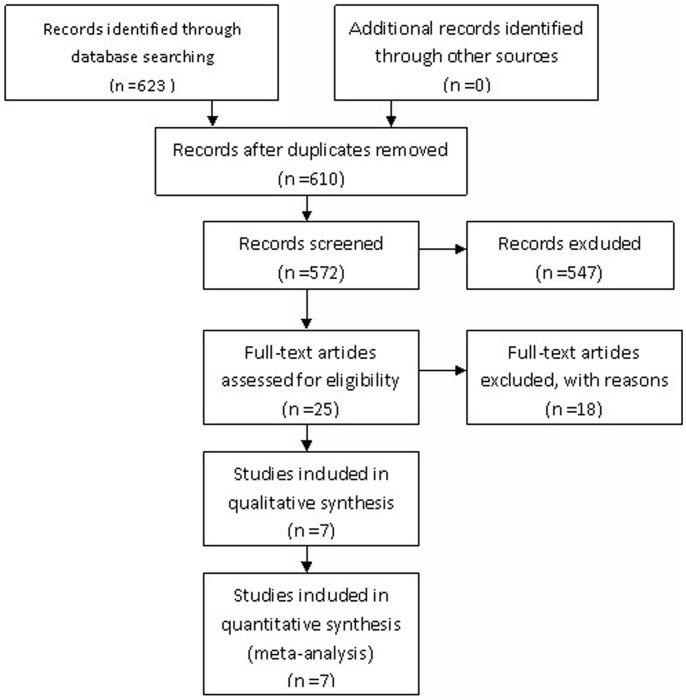
Selection of included studies.

A total of 623 study abstracts were identified through the electronic and manual searches (Figure 1). Of these, 598 did not meet criteria for inclusion in the meta-analysis. Full text was retrieved for 25 studies. On review of these studies, 18 were eliminated because they did not meet the criteria for inclusion in the meta-analysis. Of these trials, only seven trials met the inclusion criteria [15], [16], [22]–[26].

### Description of Included Trials

Detailed description of the methods used in the included trials, including the type of chemotherapeutic drugs and type of GnRHa used, is provided in Table 1. The results of quality assessment for the included studies are presented in Table 2.

**Table 1:** Description of Included Trials.

Study	Method	Participants	Intervention	Outcome
Badawy et al.	Design: Randomized trial Method of randomization: Unclear Allocation concealment: Sealed, dark envelopes Blinding: NA Sample size calculation: Performed a priori	Country: Egypt No. of participants GnRH: 40; Control: 40Age: mean ± SD [years] GnRH: 30.00±3.51 Control: 29.20±2.93 Baseline FSH: mean± SD (mIU/mL) GnRH: 4.3±1.11 Control: 5.7±1.30 Mean follow-up (y) GnRH-a: 0.66; Control: 0.66	Surgery: Modified radical Mastectomy or breast-conserving surgery plus full axillary lymph node dissection Chemotherapy: Up to 6 cycles of FAC regimen Radiotherapy: Not used GnRH: Goserelin (3.6 mg)/28 days for 6 months Control: No GnRH analog	Definition of POF: Early cessation of menstruation, ovulation and increased serum FSH level (hypergonadotropic amenorrhea) Available outcomes: Incidence of spontaneous menstruation Incidence of spontaneous ovulation
Gerber et al	Design: Randomized trial Method of randomization: NA Allocation concealment: NA Blinding:NA Sample size calculation: Performed a priori	Country: Germany No. of participants GnRH: 30; Control: 30 Age: median (range) [years] GnRH: 35.1 (26–44) Control: 38.2 (29–47) Baseline FSH: mean ± SD (mIU/mL) GnRH: NA; Control: NA Mean follow-up (y) GnRH-a: 0.5; Control: 0.5	Surgery:NA Chemotherapy: Up to 6 or 8 cycles of an Anthracycline and cyclophosphamide Radiotherapy:NA GnRH: Goserelin (3.6 mg)/28 Days until the end of chemotherapy Control: No GnRH analog	Definition of POF: Cessation of menstruation Available outcomes: Incidence of spontaneous menstruation, Incidence of spontaneous pregnancy
Del Mastro et al.	Design: Randomized trial Method of randomization: Permuted blocks Allocation concealment: Central randomization Blinding: Open trial with no Blinding Sample size calculation: NA	Country: ItalyNo. of participants: GnRH: 148; Control: 133 Age: median (range) [years] GnRH: 39(24–45) Control: 39(25–45) BaselineFSH:median(range) (mIU/mL) GnRH: 5.45(3.79–8.08) Control: 5.01(3.66–7.35) Mean follow-up (y) GnRH-a: 1; Control: 1	Surgery: NA Chemotherapy: Up to 6 cycles of CMF regimen ± tamoxifen Radiotherapy: NA GnRH: Triptorelin (3.75 mg)/28 days until the end of chemotherapy Control: No GnRH analog	Definition of early menopause: no resumption of menstrual activity and postmenopausal levels of follicle-stimulating hormone (FSH) for 1 year after the end of chemotherapy Available outcomes: the incidence of chemotherapy-induced early menopause
Munster et al.	Design: Randomized trial Method of randomization: computerized randomization Allocation concealment: NA Blinding:NA Sample size calculation: NA	Country: Unclear No. of participants GnRH: 27; Control: 22 Age: median (range) [years] GnRH: 39(21–44) Control: 38(26–44) Baseline FSH: mean ± SD (mIU/mL) GnRH:NA; Control:NA Mean follow-up (y) GnRH-a: 2; Control: 2	Surgery: NA Chemotherapy: AC (four cycles) or AC (four cycles) followed by taxane (four cycles) or FEC/FAC (six cycles) ± tamoxifen Radiotherapy: NA GnRH: Triptorelin (3.75 mg)/28 days for the duration of chemotherapy Control: No GnRH analog	Definition of Maintained or uninterrupted menses: the continuation of regular menses 21 to 35 days apart with at least 2 days of bleeding. Resumed menses required resumptions of at least three menses in 6 months and FSH level of less than 40 mIU/mL. Available outcomes: Incidence of the resumed menses
Li Mingyi et al.	Design: Randomized trial Method of randomization: NA Allocation concealment: NA Blinding:NA Sample size calculation: NA	Country:China No. of participants: GnRH: 31; Control: 32 Age: median (range) [y] GnRH: 40(21–49) Control: 39(25–50) Baseline FSH: mean ± SD (mIU/mL) GnRH: NA; ControlB: NA Mean follow-up (y) GnRH-a: NA; Control: NA	Surgery: NA Chemotherapy: AC (four cycles) or AC (four cycles) followed by taxane (four cycles) Radiotherapy: NA GnRH: Goserelin (3.6 mg)/28 days for the duration of chemotherapy Control: No GnRH analog	Definition of POF: Cessation of menstruation Available outcomes: Incidence of spontaneous menstruation
Sun Jingbo et al.	Design: Randomized trial Method of randomization:NA Allocation concealment:NA Blinding:NA Sample size calculation: NA	Country: China No. of participants: GnRH: 11; Control: 10 Age: median (range) [years] GnRH: 38(23–49) Control: 37(21–48) Baseline FSH: mean ± SD (mIU/mL) GnRH:NA; ControlB: NA Mean follow-up (y) GnRH-a: NA; Control: NA	Surgery: NA Chemotherapy: Up to 6 cycles of FEC or TE Radiotherapy:NA GnRH: Goserelin (3.6 mg)/28 days for 2 years Control: No GnRH analog ± tamoxifen	Definition of POF: Cessation of menstruation Available outcomes: Incidence of Spontaneous menstruation
Sverrisdottir et al.^a^	Design: Randomized trial Method of randomization: Permuted blocks Allocation concealment: Central randomization Blinding: NA Sample size calculation: NA	Country: Sweden No. of participants: GnRH A: 29; Control A: 28 GnRH B: 37; Control B: 29 Age: median (range) [y] GnRH A: 45(36–51) GnRH B: 46(35–54) Control A: 45(29–53) Control B: 45(29–53) Baseline FSH: mean ± SD (mIU/mL) GnRH A: NA Control A: NA GnRH B: NA Control B: NA Mean follow-up (y) GnRH-a: 1.0; Control: 1.0	Surgery: breast conserving Chemotherapy: Up to 6 cycles of CMF regimen ± tamoxifen Radiotherapy: Performed in patients with breast conserving surgery and/or four or more positive lymph nodes GnRH: Goserelin (3.6 mg)/28 days for 2 years Control: No GnRH analog ± tamoxifen	Definition of POF: Cessation of Menstruation Available outcomes: Incidence of spontaneous menstruation

Note: CMF = cyclophosphamide, methotrexate, and 5-fluorouracil regimen; FAC = 5-fluorouracil, doxorubicin, and cyclophosphamide regimen; FSH =  follicle-stimulating hormone; GnRH = gonadotropin-relea sing hormone; NA =  information not available; POF = premature ovarian failure; ^a^This study has four arms: arm A received only chemotherapy ± GnRH analogue; arm B received chemotherapy+ tamoxifen ± GnRH analogue.

**Table 2:** The Assessment Of Study Quality.

Study	Random method	Allocation concealment	Blinded	Completeness of follow-up	Score	Rating
Badawy [15]	2	1	0	0	3	B
Sverrisdottir [23]	2	1	0	2	5	A
Gerber [22]	1	0	0	2	3	B
Del Mastro [24]	2	1	0	2	5	A
Munster [16]	2	1	0	2	5	A
Li Mingyi [25]	1	0	0	0	1	C
Sun Jingbo [26]	1	0	0	0	1	C

### Meta-Analysis

**Figure 2:**
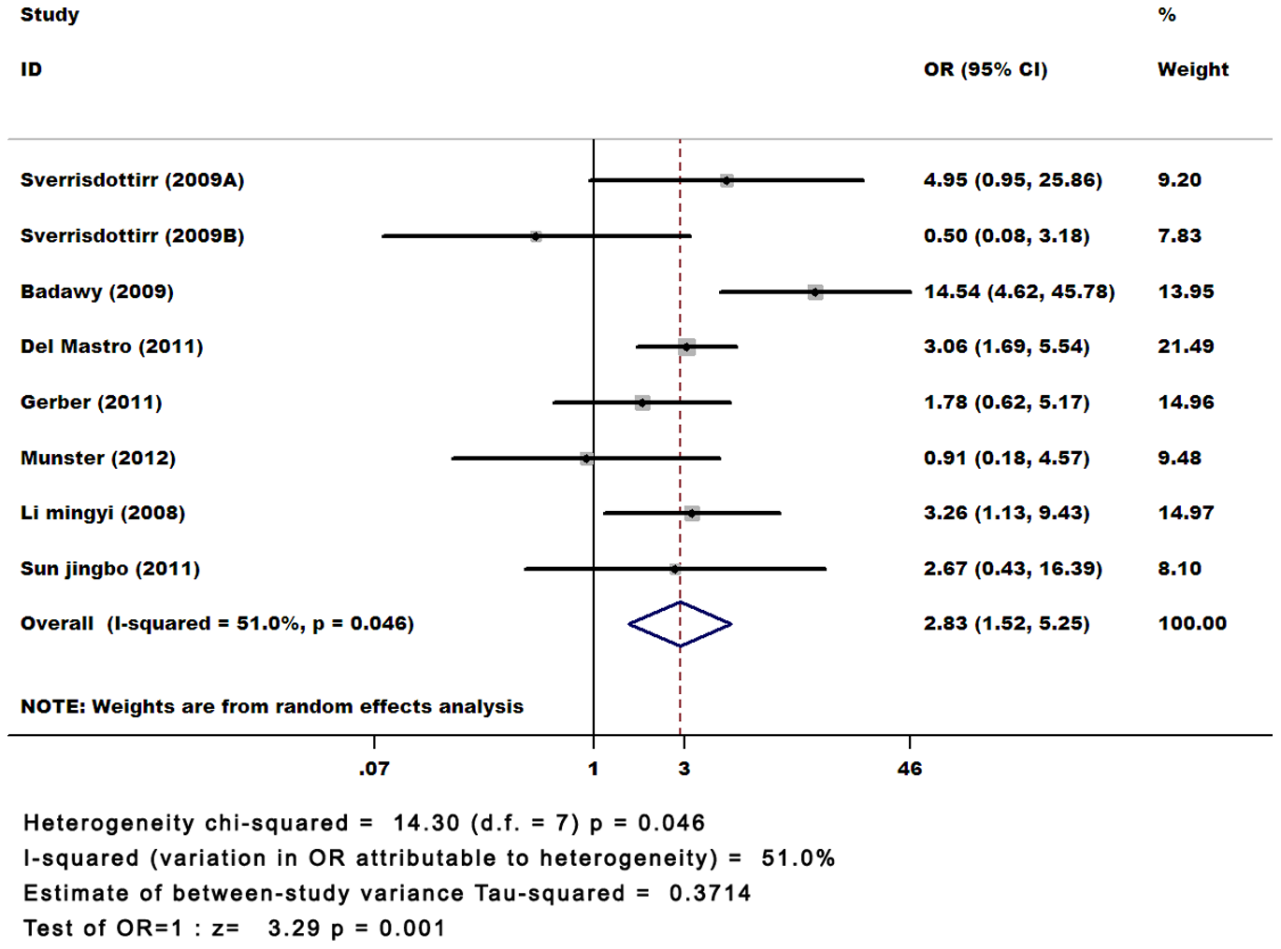
Forest plot of effect sizes for the incidence of women with spontaneous menstruation.

Through establishing strict, unified inclusion and exclusion criteria, and keeping consistent the relevant factors, we ensured clinical homogeneity of the included studies to a certain extent. However the tests for statistical heterogeneity showed moderate heterogeneity (P = 0.046, I^2^ = 51%). This may reflect the differing patient populations, chemotherapy regimens, mean patient age, type of GnRHa used, or the length of the follow-up period in the respective trials. Therefore, we used the random effect model to calculate the effect size (OR, 95% CI). The incidence of women with spontaneous menstruation was statistically greater when GnRHa was used (OR 2.83; 95% CI, 1.52–5.25). (Figure 2).

### Sensitivity Analysis

**Figure 3:**
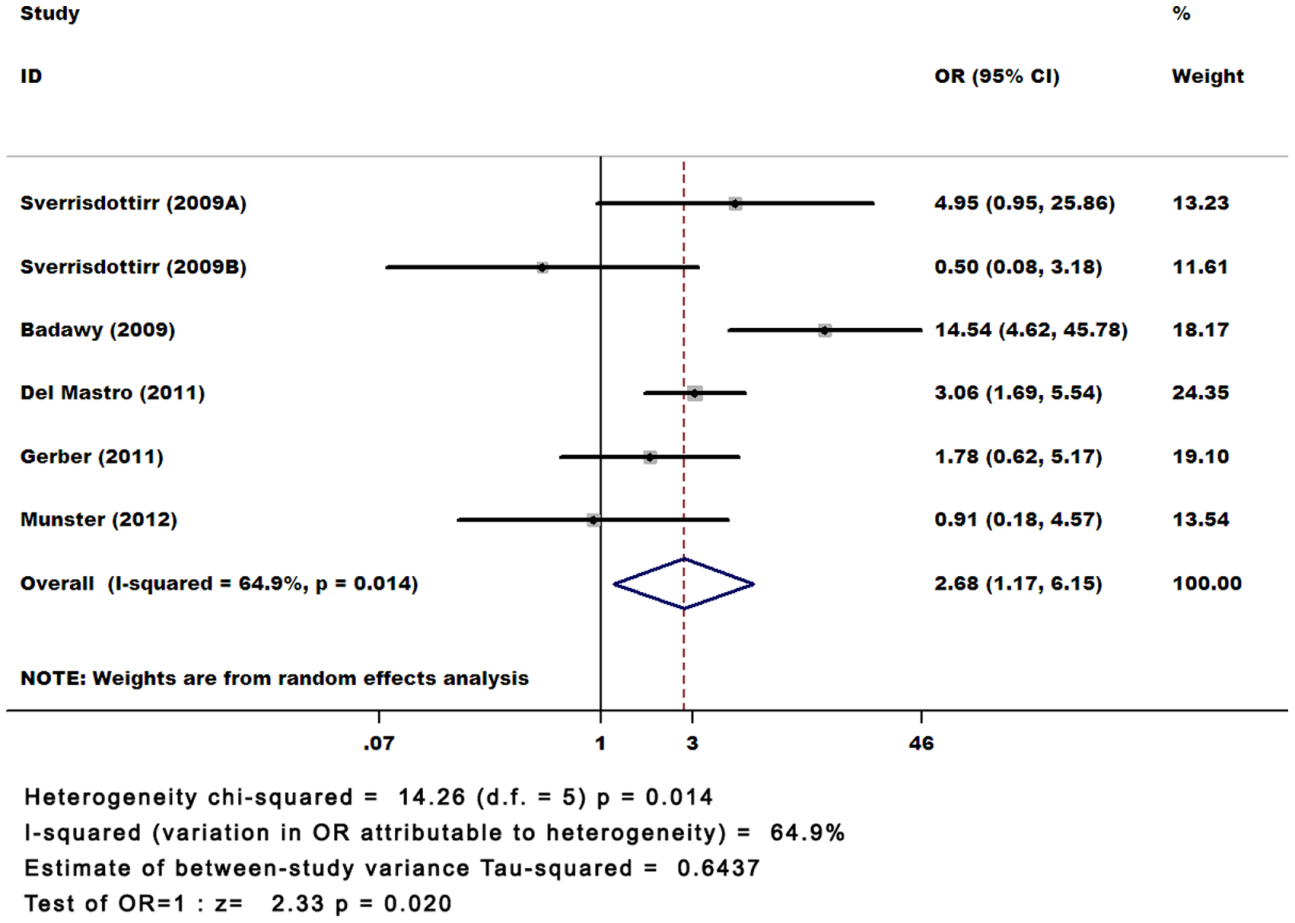
The pooled ORs for the incidence of women with spontaneous menstruation.

According to the results of the quality assessment for the included studies, we then excluded the two Chinese literature reports as being of the relatively poor quality [25], [26], and recalculated the pooled OR and 95% CI (OR 2.681; 95% CI, 1.169–6.146). The results were basically the same. (Figure 3).

### Publication Bias

**Figure 4:**
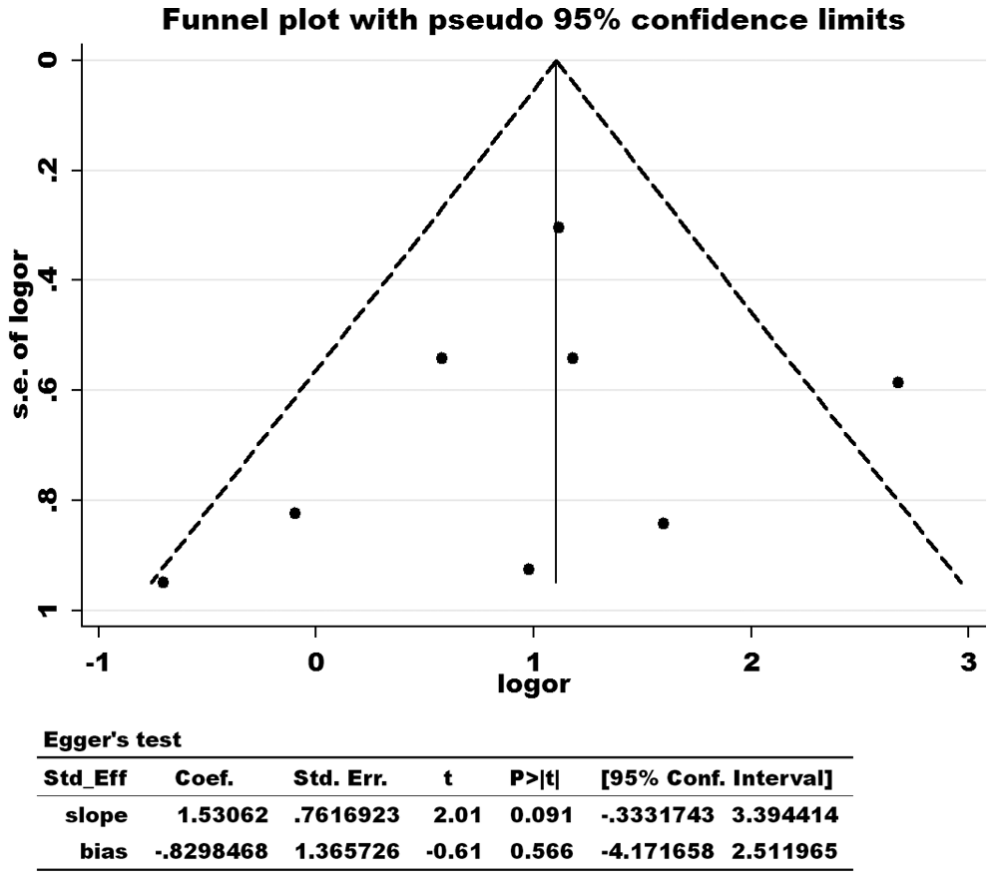
Funnel plot and Egger’s test of effect sizes for the included studies.

Funnel plots and Egger’s test were performed to assess the publication bias of the literature. Examination of the funnel plot of effect sizes by their SEs suggests a fairly even distribution of studies for the primary study outcome. Egger’s test further confirmed the absence of publication bias in this meta-analysis (P = 0.566, 95% CI, −4.17–2.51). (Figure 4).

## Discussion

The results of this systematic review show that the use of GnRHa treatment with chemotherapy may be beneficial in preserving future fertility in women treated with chemotherapeutic agents. The main observation of our analysis is that treatment with an agonist does seem to provide short-term return of ovulation and a menstrual cycle.

Beginning from the 1980 s, there were some studies on the role of GnRHa in the protection of chemotherapy-induced gonadal damage. However, not all studies had the same results and possible reasons could include: 1) GnRHa can reduce follicular loss in patients of different ages, but the clinical application is limited to young women with good ovarian reserve. With increasing age, female ovarian reserve gradually decreases, and the role of GnRHa in the protection of ovarian function is correspondingly weakened. In the meta-analysis, the youngest patients were those from the Badawy’s study [15] that seemed based on what is shown in the forest plot to be mainly responsible for the heterogeneity. 2) The time limits for the use of a GnRHa vary in length. For pituitary-ovarian suppression, GnRHa can stimulate the increase of gonadotropin for the short-term, the so-called "Ignition" period (about 1 to 2 weeks), and it can contribute to the primordial follicle developing into mature follicles. However, chemotherapy drugs are active against mitotically active cells, and GnRHa may make the ovaries more sensitive to chemotherapeutic drugs [27]. 3) Different GnRHa preparations act differently. Compared to short-acting GnRHa, the role of a long-acting GnRHa in pituitary-gonadal axis suppression is more lasting and stable. In the included studies, the types of GnRHa are all long-acting. 4) There were different follow-up periods in the studies. Generally, GnRHa adjuvant therapy should be maintained to the end of chemotherapy, and we should use GnRHa continually due to the metabolism of chemotherapy drugs. Therefore, two months after the completion of chemotherapy, and generally at 6-, 12-, and 18-month follow-up, we should reassess the ovarian function. However, how long we should follow-up needs further study. There is a lack of a multi-center, high-quality, large sample, homogeneous prospective randomized controlled clinical studies that could address some of these problems.

In the meta-analysis, seven randomized controlled trials were finally included. The results showed that GnRHa added to adjuvant chemotherapy has a protective effect on ovarian function in premenopausal women; in the GnRHa group compared with the control group, the pooled OR and 95% CI was significantly increased. However, there were bias factors that may affect the authenticity of the meta-analysis results. Through the funnel plot analysis the included studies had a distribution of basic symmetry, showing an inverted funnel, and Egger’s test analysis showed that the funnel plot is symmetrical. So, the publication bias of included literature is small and the potential bias has no substantive effect on the final conclusions. Similarly, in 2011, Bedaiwy also applied meta-analysis and obtained the same results [28].

This study had some limitations: 1) In the literature data retrieval stage, because of the limiting conditions, the included literature was only published literature, and language is limited to English and Chinese. The existence of language bias and unpublished literature results may affect the results of the meta-analysis. 2) In order to implement the strict inclusion and exclusion criteria, a small number of studies were retrieved. 3) In this study, the quality of the included studies was low. None mentioned the use of blinding, and some studies had no description of the method of randomization and did not mention the allocation concealment. These lower-quality studies may change the combined effect of the interventions in the meta-analysis. 4) The included articles only adopted menstrual recovery to reflect the ovarian function, the outcome measure was single. It would be best to assess more comprehensively by the combination of detection of ovarian reserve before and after treatment; such as the determination of the basis of sex hormones, cytokines, and ovarian ultrasonography. 5) Adverse reactions to GnRHa were not mentioned.

### Conclusion

Based on the results of this meta-analysis, GnRH treatment with chemotherapy in premenopausal women provides a potential benefit for ovarian function preservation, with higher rates of spontaneous resumption of menses. However, in order to further define the role and mechanism of GnRHa in ovarian function preservation, more well-designed and powered trials are needed. More data are needed with respect to real fertility preservation, and perhaps, more urgently, the safety of concomitant chemotherapy and hormone therapy when given at a potentially curable disease stage.
